# WEE1 promotes endometriosis via the Wnt/β-catenin signaling pathway

**DOI:** 10.1186/s12958-021-00844-8

**Published:** 2021-10-22

**Authors:** Liya Shi, Xue Xue, Hui Tian, Hongjuan Ye, Hui Wang, Rongxiang Wang, Yu Liu, Caixia Zhang, Qiuju Chen, Lihua Sun

**Affiliations:** 1 Department of Reproductive Medicine Center, Shanghai East Hospital, Tongji University School of Medicine, 150 Jimo Road, Pudong New Area, Shanghai, 200120 China; 2 Department of Assisted Reproduction, Shanghai Ninth People’s Hospital, Shanghai JiaoTong University School of Medicine, 639 Zhizaoju Road, Shanghai, 200011 China

**Keywords:** Endometriosis, WEE1, β-Catenin, Fibrosis

## Abstract

**Background:**

Endometriosis, the presence of active endometrial tissue outside the lining membrane of the uterine cavity, is a common disease in women of childbearing age. The ectopic endometrium has some characteristics of tumor tissue, including invasive and migratory abilities. In addition, endometriosis is associated with inflammation and reduced cellular apoptosis.

**Methods:**

Western blot analysis, qPCR, immunohistochemistry, immunofluorescence microscopy, Transwell assay, wound healing assay, and TUNEL staining.

**Results:**

Interleukin-1β (IL-1β) induced WEE1 expression in endometrial stromal cells (ESCs), suggesting that WEE1 may be upregulated during the endometriosis-induced inflammatory response. Overexpression of WEE1 in cultured ESCs promoted ESC migration while inhibiting apoptosis, whereas WEE1 knockdown reduced ESC migration while promoting apoptosis. Inhibition of WEE1 attenuates fibrosis in ESCs and female C57BL/6 J mice. This pro-fibrotic effect of WEE1 was significantly decreased by treatment with the Wnt/β-catenin inhibitor XAV939, suggesting that WEE1 acts via the Wnt/β-catenin signaling pathway.

**Conclusion:**

Our study demonstrates that WEE1 promotes ESC migration and fibrosis via the Wnt/β-catenin signaling pathway. Thus, WEE1 may serve as a potential therapeutic target for the treatment of endometriosis.

**Supplementary Information:**

The online version contains supplementary material available at 10.1186/s12958-021-00844-8.

## Introduction

Endometriosis is a common, chronic, inflammatory gynecological disease that affects approximately 10% of the female population of reproductive age [1, 2]. Characterized by the growth of endometrial-like tissue outside the uterine cavity [3], endometriosis causes chronic pelvic pain, dysmenorrhea and infertility [4]. Although endometriosis is considered to be pathologically benign, it nevertheless shares some similarities with malignant tumors including excessive proliferation, adhesion and invasion [3, 5–7].

Epithelial-mesenchymal transition (EMT), the process by which epithelial cells undergo biochemical and morphological changes to transform into mesenchymal cells [8], has been implicated in both endometriosis and tumor metastasis [9, 10]. Transformation into mesenchymal cells results in cells that have an enhanced migratory capacity, invasive ability, increased resistance to apoptosis, and elevated production of ECM components [8]. Indeed, several studies have suggested that EMT may be involved in the pathogenesis of endometriosis [5, 7, 11, 12]. The Wnt/β-catenin signaling pathway has also been implicated in the pathophysiology of endometriosis [13, 14], with aberrant activation of β-catenin reportedly enhancing the invasiveness of endometrial stromal cells (ESCs) [15].

WEE1 is a protein kinase involved in cell cycle regulation and the DNA damage response [16–20]. Overexpression of WEE1 has been reported in several malignant tumors such as hepatocellular carcinoma [21], breast cancer [22], glioblastoma [23], malignant melanoma [24] and colorectal cancer [25], while inhibition of WEE1, via small molecule inhibitors, has been shown to be a promising clinical approach in the treatment of cancer [17–20, 26–29]. Although WEE1 was recently identified as a differentially upregulated gene in the oocytes of ovarian endometriosis patients [30], virtually nothing is known about its role in the pathogenesis of endometriosis.

Here, we sought to determine whether WEE1 has a role in the development of endometriosis. We found that treatment with the inflammatory cytokine, interleukin-1β (IL-1β) induced WEE1 expression in ESCs, indicating that upregulation of WEE1 may be associated with the inflammatory response during endometriosis. In addition, we demonstrated that overexpression of WEE1 leads to increased proliferation, migration and fibrosis of ESCs via the β-catenin pathway. In a mouse model of endometriosis, the WEE1 inhibitor AZD1775 significantly reduced fibrosis in ectopic and eutopic tissues. Our findings indicate that WEE1 may be a potential therapeutic target in the treatment of endometriosis. Since clinically applicable inhibitors of WEE1 have previously been used to treat cancer [18, 28, 29], we propose that their clinical application could be extended to the treatment of endometriosis.

## Method and materials

### Cell culture

Human ESCs were purchased from the American Type Culture Collection (ATCC) Company (Manassas, VA, USA), No. BFN608006424. Mouse endometrial stromal cells were prepared by zymogen digestion and differential adhesion. In short, mouse tissues were digested by zymogen and the target cells were obtained by different centrifugation speeds. Cells were cultured in Dulbecco’s Modified Eagle’s Medium (Gibco, Grand Island, NY, USA) containing 10% fetal bovine serum (FBS; Gibco) and 100 U/ml penicillin and 100 μg/ml streptomycin (Sigma, St. Louis, MO, USA) at 37 °C in a humidified atmosphere of 5% CO_2_.

The WEE1 inhibitor, AZD1775, was purchased from MCE (MedChemExpress; Monmouth, NJ, USA) and was used at a concentration of 40 mg/kg/day in in vivo studies. The β-catenin inhibitor, XAV939, was obtained from MCE and used at a concentration of 10 μM in in vitro studies. IL-1β was obtained from Sigma, and was used at concentrations of 5 ng/ml and 10 ng/ mL in cultured ESCs.

### Quantitative real-time PCR (qRT-PCR)

Total RNA was extracted from mouse endometrial tissue and cultured ESCs using TRIzol (Invitrogen, Carlsbad, CA, USA) according to the manufacturer’s protocol. cDNA was generated and qRT-PCR was carried out using the SYBR Green Realtime PCR Master Mix (TOYOBO) and ABI 7500 Real-Time PCR system (Applied Biosystems, USA). The following primers, obtained from Sangon Biotech (Shanghai, China), were used:

**Table:** 

	Forward	Reverse
WEE1	TGGTGGGAGTTTAGCTGACG	GCATTTGGGATTGAGGTTCGA
α-SMA	ACCCACAATGTCCCCATCTA	TCTCCAGGGAAGAAGACGAA
Collagen I	GGGAGATGCTGGTCCTGCT	GCACCATCATTTCCACGAGC
GAPDH	ACAGCAACAGGGTGGTGGAC	TTTGAGGGTGCAGCGAACTT

GAPDH was used as the control. The relative mRNA expression was determined from three independent experiments and was calculated by the 2^−ΔΔCT^ method.

### Lentivirus construction and transduction

For WEE1 overexpression studies, full-length WEE1 was amplified using the following primers: CGGGATCCATGAGCTTCCTGAGCCGAC (B*amH* I) and CGGAATTCTC AGTATATAGTAAGGCTG (E*coR* I), then cloned into the pcDNA3.1(+) vector. The empty pcDNA3.1(+) vector was used as the control. For WEE1 knockdown experiments, the short hairpin RNA (shRNA) targeting WEE1 (5′-GGCUGGAUGG AUGCAUUUAUU-3′) was cloned into the pcDNA3.1(+) vector. Scrambled shRNA (5′-UACAUCGAUCGUGUCACGU-3′) was used as the control. The WEE1 overexpression plasmid or WEE1 shRNA were transfected into 293 T packaging cells using Lipofectamine 3000 (Invitrogen) according to the manufacturer’s protocol. After 48 h, virus particles were concentrated by centrifugation and stored at − 80 °C.

ESCs were transduced with adenovirus as described previously [31]. Briefly, ESCs were grown to 80% confluence in 6-well plates, then infected with 100 multiplicity of infection (MOI) virus for 6 h. The media was then replaced and cells were cultured for a further 24 h. The cell infection efficiency was determined by fluorescence microscopy.

### Western blot

Cultured ESCs and mouse endometrial tissue samples were lysed in RIPA lysis buffer (Abcam, Cambridge, UK). Protein concentrations were determined using a BCA protein quantitative kit (Beyotime, Shanghai, China). Samples were separated by SDS-PAGE, then transferred to polyvinyl difluoride (PVDF) membranes (Roche, Basel, Switzerland). Membranes were blocked with 5% skimmed milk in Tris-buffered saline containing 0.05% Tween 20 for 2 h at room temperature, washed with 1 × TBST, then incubated with primary antibodies against WEE1 (1:1000, Abcam), β-catenin (1:5000, Abcam), GAPDH (1:5000, Abcam), α-smooth muscle actin (α-SMA;1:5000, Abcam), and Collagen I (1:2000, Abcam) overnight at 4 °C. Membranes were washed with1 × TBST and incubated in HRP-conjugated IgG secondary antibody (1:1000, Abcam) for 1 h at room temperature. After washing again, protein bands were visualized using enhanced chemiluminescence and quantified using ImageJ. Protein bands were normalized against the GAPDH bands.

### Immunofluorescence

ESCs were fixed in 4% paraformaldehyde at room temperature for 15 min. Samples were then washed three times with PBS (5 min), incubated in 0.5% Triton X-100 for 10 mins, washed with PBS and blocked in 5% BSA for 1 h. Next, samples were incubated with primary antibodies against α-SMA (1:200, Abcam) or Collagen I (1:200, Abcam) at 4 °C overnight. Samples were washed in PBS the next day, followed by incubation in the dark with the fluorescent-labeled secondary antibody at room temperature for 1 h. After washing a further three times with PBS, nuclei were stained with DAPI and mounted. Samples were examined by fluorescence microscopy. The experiment was carried out in triplicate with 3 independent cell samples.

### Transwell assay

6-well Transwell chambers (Millipore, Massachusetts, UA) were used to examine the migration and invasion of human ESCs. Briefly, 2.5 × 10^4^ cells/well were incubated in Transwell chambers in serum-free media overnight. Media supplemented with bovine serum albumin (BSA) (10 g/L) was added to the lower wells to act as a chemoattractant for 48 h at 37 °C in an atmosphere of 5% CO_2_. The chambers were removed and cells that had migrated to the lower wells were washed with PBS, fixed with methanol at 4 °C, and stained with 0.1% Crystal Violet. Images of the stained samples were taken from five different fields of view using a 200× objective. Each experiment was performed in triplicate.

### Wound healing assay

Human ESCs were grown to 90–95% confluency in 6 well plates. A linear wound was scratched through the monolayer using a pipette tip. Floating cellular debris was removed and the media was replaced with media containing either empty, WEE1-overexpressing, scramble shRNA or shWEE1 plasmids. Cells were cultured for a further 48 h. Migration at the wound site was observed using an inverted microscope.

### Terminal deoxynucleotidyltransferase-mediated nick end labelling assay (TUNEL)

Cell apoptosis was evaluated using a TUNEL assay kit (Sangon, Shanghai, China), according to the manufacturer’s instructions. Briefly, paraffin-embedded tissue sections were fixed using 4% paraformaldehyde, specimens were carried on slides, and counterstained with 4′,6-diamidino-2-phenylindole (DAPI) for nuclear localization, without permeabilization. The cells were visualized by fluorescence microscopy and apoptotic cells were marked in both red (fragmented DNA) and blue (nuclear DNA). The experiment was carried out in triplicate.

### Immunohistochemistry (IHC)

Mouse endometrial tissue samples were fixed in formalin, dehydrated and embedded in paraffin. Five micrometre thick sections were cut, deparaffinized and rehydrated. Next, sections were heated in 10 mM sodium citrate buffer (pH 6.0) at 95 °C for 10 min, washed three times in PBS at room temperature for 5 min, incubated in 3% H_2_O_2_ at room temperature for 10 min and finally blocked in goat serum at room temperature for 30 min. Sections were then incubated with primary antibodies against WEE1 (1:500, Abcam), α-SMA (1:100, Abcam) or Collagen I (1:100, Abcam) at 4 °C overnight. After washing in PBS, sections were incubated with goat polyclonal secondary antibody (1:100, Abcam) at room temperature for 1 h. Finally, sections were washed in PBS, stained with diaminobenzidine (DAB), counterstained with haematoxylin, dehydrated and mounted. Samples were observed by light microscopy. Masson’s trichrome staining was also carried out to detect fibrosis.

### Animal experiments

Female, C57BL/6 J mice (6–8 weeks old) were obtained from Shanghai Slac Laboratory Animal Co. (China). Mice were housed in a facility at 25 °C with a 12 h light/dark cycle and 50–60% humidity. All animal experiments were carried out in accordance with the ethical guidelines of LongHua Hospital, Shanghai University of Traditional Chinese Medicine.

Forty mice were randomly assigned to four groups (10 mice/group). Control mice were not subjected to estrogen or WEE1 inhibitor treatment. The estrogen group consisted of mice treated with Estradiol (100 μg/kg/week; Sigma). The AZD1775 group consisted of mice treated with the WEE1 inhibitor, AZD1775 (40 mg/kg/day; MCE), while the AZD1775 + estrogen group consisted of mice treated with AZD1775 (40 mg/kg/day) and Estradiol (100 μg/kg/week).

The drug was injected through the abdominal cavity at 10 a.m., after 1 week of drug injections, endometriosis was surgically induced in intact mice under aseptic conditions as described previously [32]. An incision (0.5 cm) was made in the abdomen to expose the uterus. One uterine horn was removed, placed in saline and cut into small fragments (1 mm^3^), then injected back into the abdominal cavity of the same mouse. The incision was sutured layer by layer. Mice were sacrificed 3 weeks after surgery.

### Statistical analysis

Statistical analysis was performed using GraphPad Prism software. Data are presented as mean ± SEM. Each experiment was performed at least three independent times. One-way ANOVA in combination with Tukey’s test was carried out to compare the multiple groups (three to four groups), while Student’s *t*-test was used to compare two groups. *P* < 0.05 was statistically significant.

## Results

### WEE1 is up-regulated in ESCs under IL-1β-induced inflammation

**Fig. 1:**
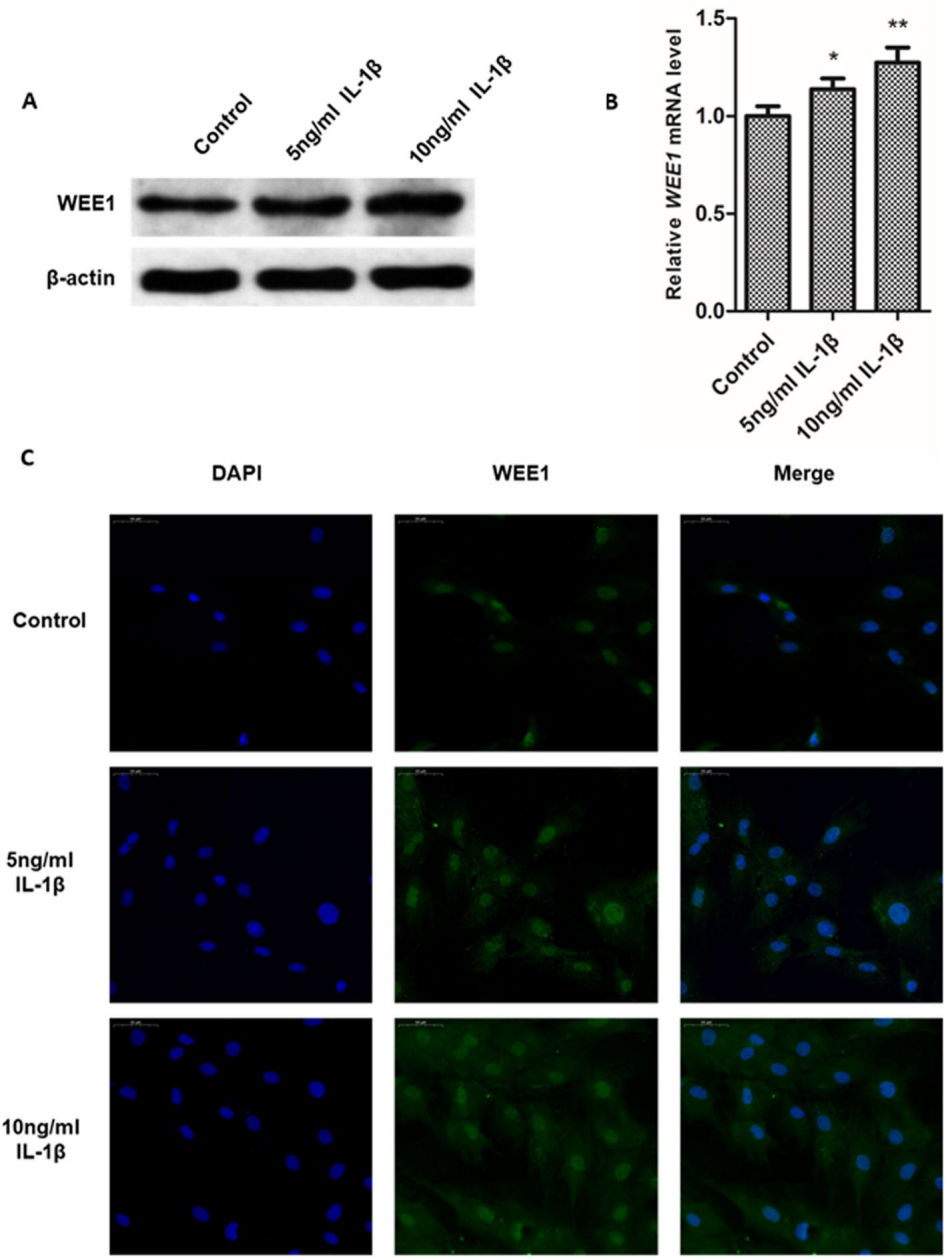
WEE1 expression increased in ESCs under inflammatory conditions. **A**, **B** Western blot and qPCR were used to detect the expression levels of WEE1 protein (**A**) and mRNA (**B**) in ESCs treated with 5 ng/ml and 10 ng/ mL IL-1β. Relative WEE1 mRNA levels are presented as mean ± SD. **C** Representative immunofluorescence images showing WEE1 expression in ESCs treated with 5 ng/ml and 10 ng/ mL IL-1β, respectively. Scale bar: 50 μm. * *p* < 0.05; ** *p* < 0.01 compared to control cells

Studies have shown that WEE1 is significantly upregulated in oocytes of endometriosis patients [30]. Moreover, the microenvironment of endometriosis is accompanied by a significant inflammatory response [33]. Here, we treated human ESCs with the inflammatory cytokine IL-1β to detect changes in WEE1 expression during the inflammatory response. Our western blot and qPCR data (Fig. 1A, B) revealed that the expression of WEE1 significantly increased with increasing IL-1β concentrations. Similarly, immunofluorescence staining (Fig. 1C) also demonstrated that IL-1β treatment led to an increase in WEE1 expression in human ESCs. Thus, taken together, our findings demonstrated that WEE1 expression was increased during the inflammatory response. Since endometriosis is accompanied by inflammation in humans, our results suggest that WEE1 expression levels may be due to the endometriosis-induced inflammatory response.

### WEE1 promotes migration and inhibits apoptosis of ESCs

**Fig. 2:**
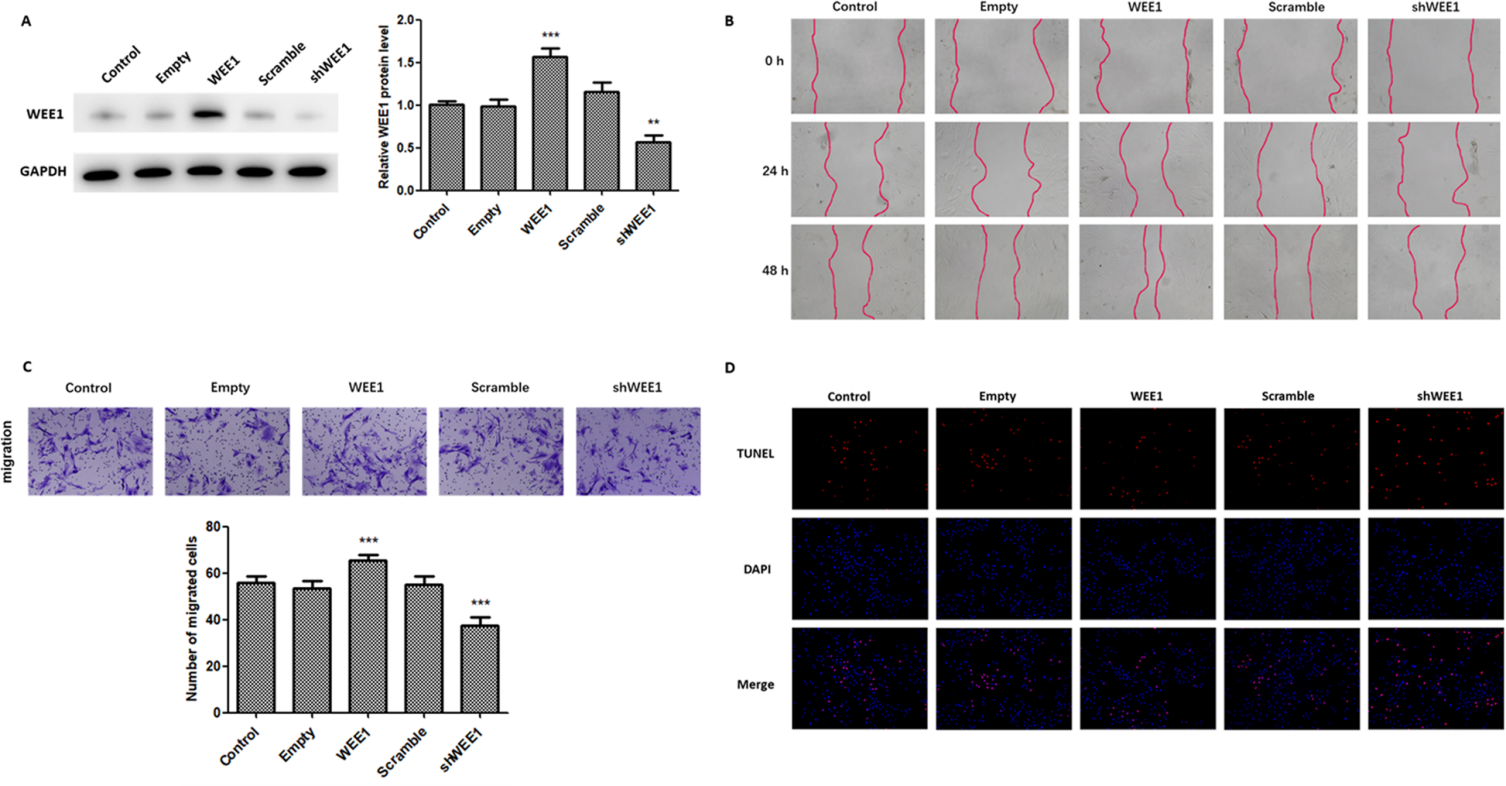
WEE1 promotes ESC migration and limits apoptosis in ESCs. **A** Western blot analysis of WEE1 protein levels in WEE1 overexpressing or knockdown ESCs. WEE1 protein levels were normalized against β-actin levels, and quantitative analysis of protein content was performed using ImageJ. Relative WEE1 protein levels are presented as mean ± SD. **B** The wound healing assay was used to examine the effects of WEE1 overexpression and knockdown on ESC migration. Scale bar: 200 μm. **C** The Transwell assay was used to determine the effect of WEE1 overexpression and knockdown on ESC migration. Scale bar: 50 μm. **D** The TUNEL assay was performed to examine the role of WEE1 overexpression and knockdown on the apoptosis of ESCs. Scale bar: 200 μm. ** *p* < 0.01; *** *p* < 0.001 compared to control cells

To examine the role of WEE1 during the development of endometriosis, we first sought to determine the effects of WEE1 overexpression or knockdown on the migration of ESCs in vitro. As shown in Fig. 2A, WEE1 expression was significantly higher in cells transfected with a WEE1-overexpression plasmid, while WEE1 levels were significantly decreased after treatment with shWEE1. Both the wound healing (Fig. 2B) and Transwell assays (Fig. 2C) revealed that ESC migration was significantly increased in cells overexpressing WEE1, but significantly lower after WEE1 knockdown, suggesting that WEE1 has a role in mediating ESC migration.

Since recent studies have demonstrated that inhibition of WEE1 can lead to mitotic catastrophe and apoptosis in cancer cells [26, 28], we next examined the effects of WEE1 overexpression and knockdown on apoptosis in ESCs. TUNEL staining revealed that knockdown of WEE1 led to increased levels of apoptosis, whereas WEE1 overexpression resulted in fewer apoptotic cells (Fig. 2D). These experimental data suggest that WEE1 can promote the migration and survival of ESCs.

### WEE1 elevates fibrosis in ESCs

**Fig. 3:**
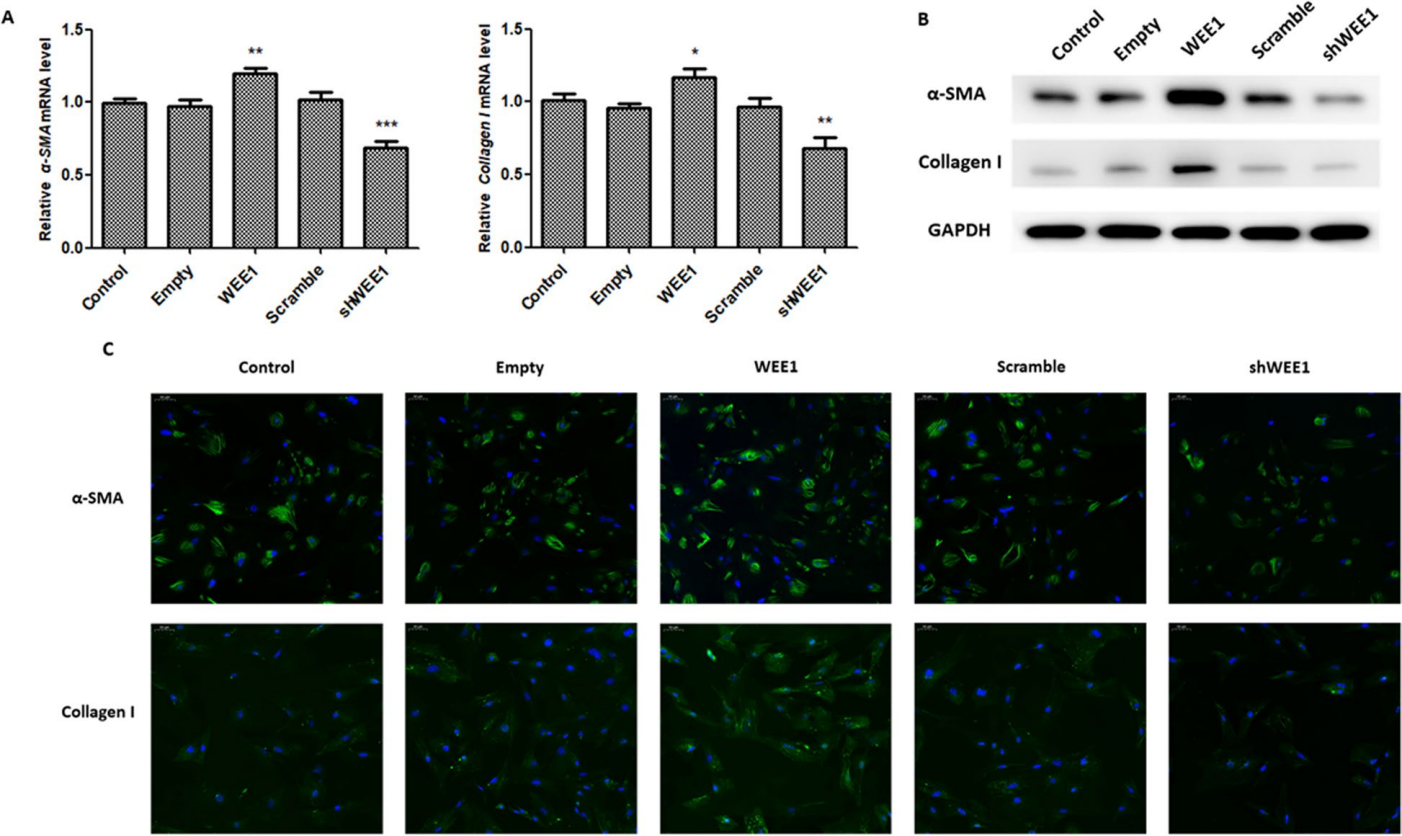
WEE1 promotes fibrosis in ESCs. **A** qPCR analysis of α-SMA and Collagen I mRNA levels in WEE1 overexpressing or knockdown ESCs. Relative α-SMA and Collagen I mRNA levels are presented as mean ± SD. **B** Western blot analysis of α-SMA and Collagen I protein levels in WEE1 overexpressing or knockdown ESCs. **C** Representative images showing immunofluorescence staining of α-SMA and Collagen I expression in WEE1 overexpressing or knockdown ESCs. Scale bar: 50 μm. * *p* < 0.05; ** *p* < 0.01; *** *p* < 0.001 compared to control ESCs

The role of WEE1 during fibrosis was examined in ESCs using Collagen I and α-SMA, as markers for tissue fibrosis [34]. As shown in Figs. 3A-B, α-SMA and Collagen I mRNA (Fig. 3A) and protein (Fig. 3B) expression levels were significantly increased in ESCs overexpressing WEE1, while knockdown of WEE1 led to a significant decrease. Immunofluorescence microscopy revealed that α-SMA and Collagen I were localized to the cytoplasm (Fig. 3C). Furthermore, increased α-SMA and Collagen I staining was observed in WEE1-overexpressing cells compared to WEE1 knockdown ESCs. Taken together, these findings suggest that WEE1 elevates fibrosis in ESCs.

### Inhibition of WEE1 prevents endometrial fibrosis in mice

**Fig. 4:**
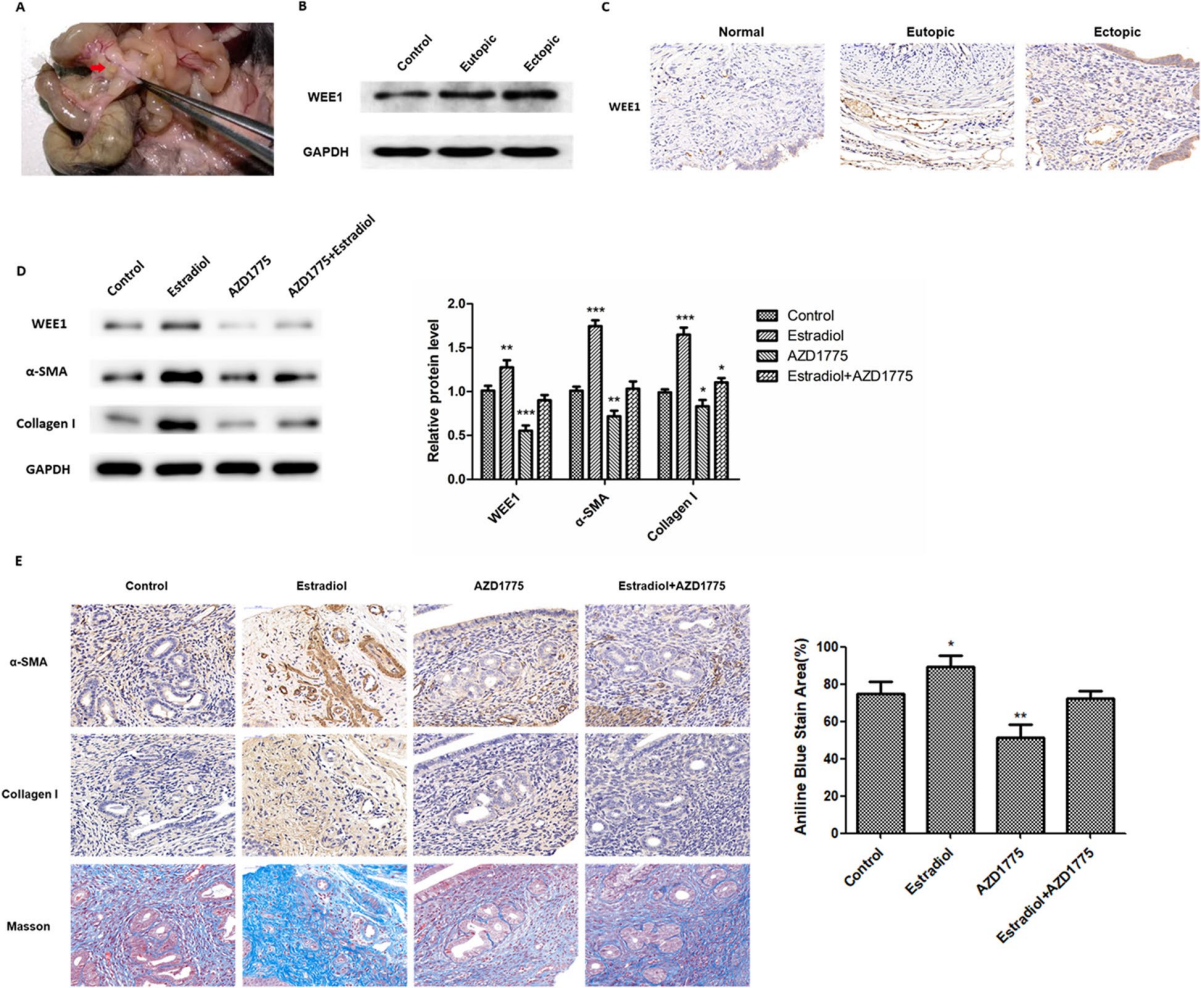
Inhibition of WEE1 prevents endometrial fibrosis in mice. **A** Representative abdominal findings from control mouse 3 weeks after induction of endometriosis. Endometriotic lesions are mainly observed as small cysts. **B** Protein levels of WEE1 in eutopic and ectopic endometrial tissues from a mouse model of endometriosis compared with normal mouse endometrial tissues by western blotting. **C** Expression of WEE1 in eutopic and ectopic endometrial tissues from a mouse model of endometriosis and in endometrial tissues from control mice was assessed by immunohistochemistry. **D** Western blot analysis of WEE1, α-SMA and Collagen I protein expression in the uterine tissue from control, Estradiol-, AZD1775- or Estradiol+AZD1775-treated mice 3 weeks after induction of endometriosis. **E** Representative immunohistochemistry images showing α-SMA, Collagen I and Masson’s trichrome staining in the uterine tissue of control, Estradiol-, AZD1775- or Estradiol+AZD1775-treated mice 3 weeks after induction of endometriosis. Quantitative analysis of percentage of Aniline Blue stained tissue after Masson’s trichrome staining. Data are presented as mean ± SD. Scale bar: 50 μm. * *p* < 0.05; ** *p* < 0.01; *** *p* < 0.001 compared to control tissue

We next sought to determine whether the fibrosis-promoting effects of WEE1 observed in vitro in ESCs also occurred in vivo in our mouse model of endometriosis. As shown in Fig. 4A, small endometriotic lesions were observed in the abdomen of endometriosis-induced mice. Prior to induction of endometriosis, mice were treated with either Estradiol (100 μg/kg/week), the WEE1 inhibitor, AZD1775 (40 mg/kg/day) or Estradiol+AZD1775. Using western blotting to examine the eutopic and ectopic endometrium of the endometriosis mouse model, we found significantly increased WEE1 expression in the eutopic and ectopic endometrium compared to the endometrium of normal mice (Fig. 4B). Immunohistochemical staining also showed that the expression of WEE1 was significantly increased in eutopic and ectopic endometrium (Fig. 4C). In addition, western blot analysis of endometrial tissue revealed that treatment with estrogen resulted in increased protein expression of WEE1, α-SMA and Collagen I indicative of increased endometrial fibrosis (Fig. 4D). However, treatment with the WEE1 inhibitor, AZD1775, led to a reduction in WEE1, α-SMA and Collagen I protein expression, suggesting that inhibition of WEE1 could reduce the level of endometrial fibrosis (Fig. 4D). Immunohistochemical staining of mice uteri from the different treatment groups revealed that treatment with the WEE1 inhibitor led to decreased α-SMA and Collagen I staining, consistent with reduced endometrial fibrosis (Fig. 4E). Masson’s trichrome staining revealed significantly less collagen in the uteri of AZD1775-treated mice (Fig. 4E). Taken together, our results demonstrated that WEE1 expression is significantly upregulated in a mouse model of endometriosis, and that inhibition of WEE1 can prevent the development of endometriosis-induced endometrial fibrosis in mice.

### WEE1 promotes activation of the β-catenin signaling pathway in ESCs

**Fig. 5:**
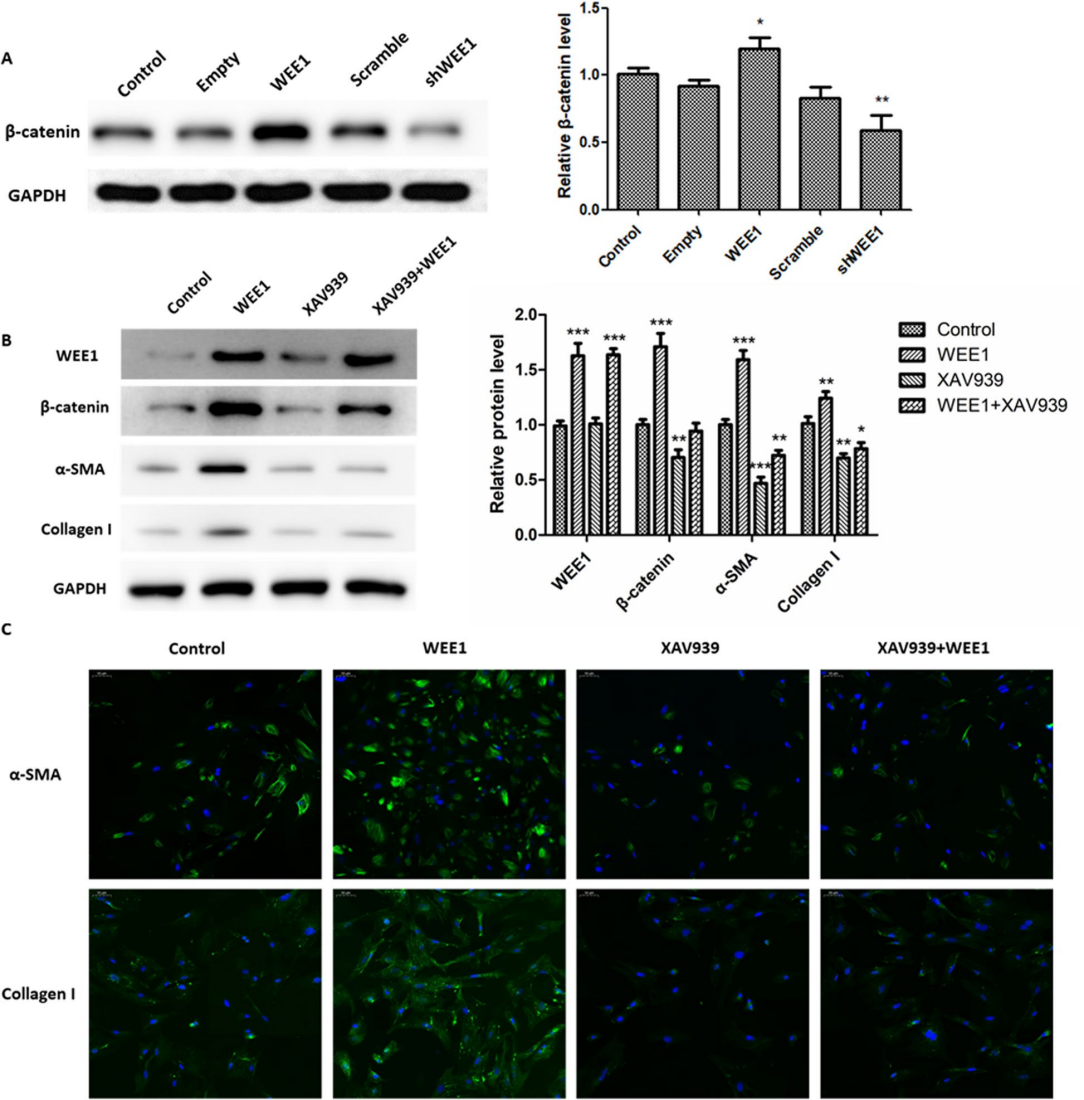
WEE1 promotes activation of the Wnt/β-catenin signaling pathway in ESCs. **A** Western blot analysis of β-catenin protein levels in WEE1 overexpressing or knockdown ESCs. Relative β-catenin protein levels are presented as mean ± SD. **B** Western blot analysis of WEE1, β-catenin, α-SMA and Collagen I protein levels in WEE1-overexpressing ESCs treated with the selective Wnt/β-catenin inhibitor, XAV939. Relative protein levels are presented as mean ± SD. **C** Immunofluorescence was used to detect the expression of α-SMA and Collagen I in ESCs. Scale bar: 50 μm. * *p* < 0.05; ** *p* < 0.01; *** *p* < 0.001 compared to control cells

Since the Wnt/β-catenin signaling pathway has been implicated in the pathophysiology of endometriosis [13, 35], we next examined whether WEE1 exerts its fibrosis-inducing effects in ESCs via the β-catenin signaling pathway. We examined β-catenin protein expression levels in WEE1 overexpressing or knockdown ESCs, and found that WEE1 overexpression led to a significant increase in β-catenin, while WEE1 knockdown resulted in a significant decrease in β-catenin expression (Fig. 5A). These findings suggest that WEE1 may act via the Wnt/β-catenin pathway. We next treated WEE1-overexpressing ESCs with XAV939, a selective inhibitor of Wnt/β-catenin-mediated transcription, to determine whether WEE1-induced fibrosis was mediated through the Wnt/β-catenin signaling pathway. As shown in Fig. 5B, α-SMA and Collagen I protein levels were significantly decreased after treatment with XAV939, even in cells overexpressing WEE1. However, XAV939 treatment had no significant effect on WEE1 protein level. This suggests that WEE1 promotes fibrosis by promoting β-catenin expression. In addition, immunofluorescence staining revealed that α-SMA and Collagen I expression was significantly decreased in ESCs after XAV939 treatment (Fig. 5C). Furthermore, a significant XAV939-induced inhibitory effect was also observed in ESCs overexpressing WEE1. Thus, taken together, our data suggest that WEE1 promotes fibrosis in ESCs through the Wnt/β-catenin signaling pathway.

## Discussion

Although endometriosis is a common gynecological disease that affects approximately 10% of reproductive age females, causing chronic pain, dysmenorrhea and infertility, the mechanisms underlying the pathogenesis of this disease remain unclear [1, 4]. To date, there is no known cure for endometriosis, and the treatment strategies currently available focus on management of the patient’s pain or infertility [36]. Thus, there is an urgent need to understand the mechanisms underlying endometriosis in the hope of developing novel and effective therapies.

Endometriosis is associated with increased inflammation and decreased apoptosis [33, 37–39]. Increased expression of inflammatory cytokines (such as IL-1α, IL-1β and TNFα) and adhesion molecules have been shown to lead to an altered peritoneal environment that promotes the adhesion, growth and survival of ectopic endometrial cells [40, 41]. Here, we used IL-1β to induce inflammation in ESCs and found that WEE1 expression was upregulated. Interestingly, WEE1 was recently identified as a differentially upregulated gene in the oocytes of ovarian endometriosis patients [30]. Thus, we hypothesized that the upregulation of WEE1 observed in endometriosis patients may be associated with the endometriosis-induced inflammatory response. Our hypothesis was subsequently validated in a mouse model of endometriosis, confirming that WEE1 appears to be involved in the pathogenesis of endometriosis.

Endometriosis shares several characteristics with malignant tumor progression including increased invasiveness and migration, as well as resistance to apoptosis and elevated ECM production [8]. WEE1 is a protein kinase that is involved in cell cycle regulation and the DNA damage response [16–20] and has been shown to be overexpressed in several types of malignant tumors [21–25]. Here, we found that overexpression of WEE1 in ESCs led to increased migration in wound healing and Transwell assays, as well as decreased apoptosis. In contrast, WEE1 knockdown reduced ESC migration and increased apoptosis. Together, these findings indicate that WEE1 is involved in ESC migration and survival, and may therefore have a role in the development of endometriosis.

The growth of endometrial tissue outside the uterine cavity leads to internal bleeding, inflammation and fibrosis [36, 40]. Here, we found that overexpression of WEE1 led to increased expression of the fibrotic markers, α-SMA and Collagen I, in both ESCs as well as in our mouse model of endometriosis, suggesting that WEE1 may also have a role in mediating fibrosis. Furthermore, since treatment with IL-1β increased WEE1 expression levels in ESCs, modulation of WEE1 in response to inflammatory signals may provide an additional strategy for limiting fibrosis during endometriosis.

The development of WEE1 inhibitors such as AZD1775 [20] has been promising in the treatment of cancer [28]. WEE1 inhibition by AZD1775 allows cells with a dysregulated G1 checkpoint to progress through the G2 checkpoint unchecked resulting in mitotic catastrophe and apoptosis [28]. In the current study, we found that treating mice with AZD1775 led to decreased fibrosis in the uteri of endometriosis-induced mice. Thus, inhibition of WEE1 prevents the development of endometrial fibrosis in vivo.

Since the Wnt/β-catenin signaling pathway has been implicated in the pathophysiology of endometriosis [13, 35], we examined whether WEE1 exerts its fibrosis-inducing effects in ESCs via the β-catenin signaling pathway. We found that overexpression of WEE1 led to an increase in β-catenin expression in ESCs. Inhibition of β-catenin with the tankyrase inhibitor XAV939 led to decreased fibrosis as measured by α-SMA and Collagen I expression. Thus, our findings suggest that WEE1 acts via the Wnt/β-catenin signaling pathway to promote fibrosis in ESCs.

## Conclusions

Our study describes a role for WEE1 in the pathogenesis of endometriosis. Our preliminary data indicate that upregulation of WEE1 as part of the endometriosis-induced inflammatory response may lead to increased fibrosis. Furthermore, we demonstrated that inhibition of WEE1 reduces endometrial fibrosis in mice. Since WEE1 inhibitors have already been used in the treatment of cancers [20, 26, 28], we propose that these inhibitors could be applied to patients with endometriosis. Future studies should further examine the effects of WEE1 inhibitors on the progression of endometriosis in patients susceptible to this disease.

## Supplementary Information

**Additional file 1.**

## Data Availability

All data in this study are included in this manuscript.

## Abbreviations

WEE1: WEE1 G2 checkpoint kinase
ECM: Extracellular matrix
EMT: Epithelial-mesenchymal transition
ESCs: Endometrial stromal cells
XAV939: Wnt/β-catenin inhibitor
AZD1775: WEE1 inhibitor
α-SMA: α-smooth muscle actin
IL-1β: Interleukin-1β
