# Supplementary figures and images for: WEE1 promotes endometriosis via the Wnt/β-catenin signaling pathway
Source: Reprod Biol Endocrinol. 2021 Oct 22;19:161. doi: 10.1186/s12958-021-00844-8 (PMC8532311; doi:10.1186/s12958-021-00844-8)

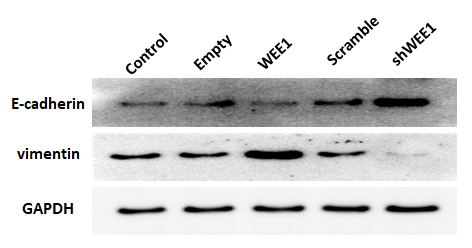

Supplement: Supplementary file 1 — Additional file 1. [file 12958_2021_844_MOESM1_ESM.tif]
